# Randomised control trial of the effectiveness of an integrated psychosocial health promotion intervention aimed at improving health and reducing substance use in established psychosis (IMPaCT)

**DOI:** 10.1186/s12888-017-1571-0

**Published:** 2017-12-28

**Authors:** Fiona Gaughran, Daniel Stahl, Khalida Ismail, Kathryn Greenwood, Zerrin Atakan, Poonam Gardner-Sood, Brendon Stubbs, David Hopkins, Anita Patel, John Lally, Philippa Lowe, Maurice Arbuthnot, Diana Orr, Sarah Corlett, Jonas Eberhard, Anthony S. David, Robin Murray, Shubulade Smith, on behalf of the IMPaCT Team

**Affiliations:** 1 National Psychosis Service, South London and Maudsley NHS Foundation Trust, London, UK; 2 Department of Psychosis Studies, Institute of Psychiatry, Psychology and Neuroscience, Kings College London, London, UK; 3 Biostatistics Department, Institute of Psychiatry, Psychology and Neuroscience, Kings College London, Denmark Hill, London, UK; 4 Department of Psychological Medicine, Institute of Psychiatry, Psychology and Neuroscience, Kings College London, Denmark Hill, London, UK; 5 Sussex Partnership NHS Foundation Trust and School of Psychology, University of Sussex, Brighton, UK; 6 Health Service and Population Research Department, Institute of Psychiatry, Kings College London, London, UK; 7 Physiotherapy Department, South London and Maudsley NHS Foundation Trust, London, UK; 8 Department of Diabetic Medicine, King’s College Hospital NHS Foundation Trust, King’s Health Partners, London, UK; 9 Health Economics, Centre for Primary Care & Public Health, Blizard Institute, Queen Mary University of London, London, UK; 10 National Psychosis Service, South London and Maudsley NHS Foundation Trust, London, UK; 11 Carer Expert and Chair of Trustees, Rethink Mental Illness, London, UK; 12 London, UK; 13 London, UK; 14 Interim Director of Public Health, London Borough Of Lambeth, London, UK; 15 Department of Clinical Sciences, Lund University, Lund, Sweden; 16 Institute of Psychiatry, Psychology and Neuroscience, Kings College London, Denmark Hill, London, UK; 17 Department of Forensic and Neurodevelopmental Science, Institute of Psychiatry, Psychology and Neuroscience, Kings College London, Denmark Hill, London, UK; 18 Forensic Intensive Care Service, South London and Maudsley NHS Foundation Trust, London, UK

**Keywords:** Mortality, Health promotion intervention, Psychosis, Quality of life, Schizophrenia

## Abstract

**Background:**

People with psychosis have a reduced life expectancy of 10–20 years, largely due to cardiovascular disease. This trial aimed to determine the effectiveness of a modular health promotion intervention (IMPaCT Therapy) in improving health and reducing cardiovascular risk in psychosis.

**Methods:**

A multicentre, two arm, parallel cluster RCT was conducted across five UK mental health NHS trusts. Community care coordinators (CC) were randomly assigned to training and supervision in delivering IMPaCT Therapy or treatment as usual (TAU) to current patients with psychosis (cluster). The primary outcome was the physical and mental health subscales of the Short form-36 (SF-36) questionnaire.

**Results:**

Of 104 care coordinators recruited, 52 (with 213 patients) were randomised to deliver IMPaCT therapy and 52 (with 193 patients) randomised to TAU. Of 406 patients, 318 (78%) and 301 (74%) attended 12- and 15-month follow-up respectively. IMPaCT therapy showed no significant effect on the physical or mental health component SF-36 scores versus TAU at 12 or 15 months. No effect was observed for cardiovascular risk indicators, except for HDL cholesterol, which improved more with IMPACT therapy than TAU (Treatment effect (95% CI); 0.085 (0.007 to 0.16); *p* = 0.034). The 22% of patients who received >180 min of IMPACT Therapy in addition to usual care achieved a greater reduction in waist circumference than did controls, which was clinically significant.

**Conclusion:**

Training and supervising community care coordinators to use IMPaCT therapy in patients with psychosis is insufficient to significantly improve physical or mental health quality of life. The search for effective, pragmatic interventions deliverable in health care services continues.

**Trial registration:**

The trial was retrospectively registered with ISRCTN registry on 23/4/2010 at ISRCTN58667926; recruitment started on 01/03/2010 with first randomization on 09.08.2010 ISRCTN58667926.

## Background

There is a wealth of evidence demonstrating that people with psychosis, including schizophrenia, schizoaffective disorder and bipolar disorder, have considerably worse physical health than the general population [1, 2]. Of particular concern are the high rates of diabetes, metabolic syndrome (MetS) and cardiovascular disease (CVD) [2]. This increased CVD risk profile is the largest risk factor for the reduction in life expectancy of between 10 and 20 years observed in people with psychosis [3, 4]. Furthermore this mortality gap is widening [3, 4].

The underlying causes of the increase in CVD and premature mortality are complex and multi-factorial. Whilst genetic factors and shared pathophysiological mechanisms contribute, unhealthy lifestyles including high rates of smoking, alcohol use disorders, poor diet, high levels of sedentary behaviour and low levels of physical activity are modifiable factors [1, 2]. In the general population, levels of CVD appear to have plateaued, but they are increasing for people with psychosis [5]. This may be in part because general public health initiatives, such as smoking cessation campaigns and calls to increase physical activity, do not effectively reach this population [6].

Our recent [7] large UK prevalence study, conducted as part of the baseline for this trial demonstrated that nearly two-thirds of people with established psychosis had MetS; similar to the 54.8% reported in the second Australian national survey of psychosis [8] with extremely high levels of central obesity. A Spanish study, using a different definition of MetS, found that patients with schizophrenia treated with antipsychotics had similar rates of MetS to people 10 to 15 years older in the general population [9, 10]. Additionally, 28% of the UK sample had “hazardous drinking” (AUDIT score ≥ 8), 23% had a high saturated fat intake [11], and on average participants spent over 8 h of their waking day being sedentary [7]. Of additional concern, 62% (*n* = 268/432) smoked tobacco, with a mean of 18 cigarettes per day, comparable to the 66.1% reported in the Australian study [7, 8].

A recent meta-analysis [12] of 17 randomised control trials (RCT) demonstrated a modest positive influence of lifestyle interventions on the prevention and reduction of weight gain and cardiometabolic risk in people with psychosis. Exercise [13] and health-mentor delivered nutritional and exercise interventions [14] also display promise. Whilst these results are encouraging, to date the majority of such interventions targeting people with psychosis have been ‘added on’ to standard care or focus on individual risk factors such as weight or BMI. Such interventions may exclude those patients not attending community mental healthcare services regularly or with difficulties in organisation or motivation. Nor do they fully allow for the multiple health risks experienced by this population. To date, few large-scale long-term RCTs have attempted to improve health in its widest sense in people with psychosis, with most concentrating on a single target, usually weight, with interventions largely run by therapists outside of the usual clinical team. Some studies have co-located the work; for instance, Daumit [15] et al. found that offering free exercise classes and healthy meals in community rehabilitation centres successfully reduced weight in people with psychosis, but again these were separate from usual mental health care and the intervention involved a high level of intense input. Moreover, the recent In SHAPE study [16] in the US relied upon attendance at a public fitness club, which can prove a barrier for some people with psychosis without adequate support. A well-designed large RCT in Denmark, running in parallel with IMPaCT, evaluated an intense intervention of add-on multifactorial lifestyle coaching and additional care co-ordination delivered in the patient’s usual healthcare setting but recently reported that this did not reduce cardiovascular risk [17]. Moreover, like much of the other work to date it involved significant additional input, which will have cost implications. The evidence-base therefore is not yet sufficient to plan services. There is a need for effective and cost-effective ways to reduce cardiovascular risk in a way that is accessible to all patients in community mental health services.

We therefore used the emerging knowledge base to design the content of a novel, manualised, modular health promotion intervention (HPI) to manage physical health and substance use disorders in people with psychosis – IMPaCT Therapy [18]. We chose an over-arching primary outcome measure, quality of life, to reflect both mental and physical health states, rather than focus on one specific marker of cardiometabolic risk. We hypothesised that the integration of IMPACT therapy with usual care would be more effective than usual mental health care in improving quality of life as measured by the physical (PCS) and mental (MCS) component of the Short form (SF)-36 scale [19], at 12-months follow-up in people with psychosis. We proposed that this would be sustained 3 months after completion of the formal intervention, at 15-months follow up. We also hypothesised that the addition of IMPaCT therapy to TAU would result in better cardiometabolic outcomes and healthier lifestyle/substance use choices than in those receiving TAU alone.

## Methods

### Development of the Health Promotion Intervention (IMPaCT Therapy)

We developed a novel integrated health promotion intervention (HPI), IMPaCT therapy, drawing on key principles of two existing effective interventions for physical health and substance use; the “Well-being Support Programme” [20] and “Managing Mental Health and Drug Use” [21], adapted for use in routine clinical care and implementation by the patient’s usual care coordinator. In UK secondary care community mental health services, care coordinators are the main point of clinical contact and are from multiple professions; usually mental health nursing but also social work, psychology or occupational therapy. The intervention aimed to be pragmatic enough to be deliverable within the National Health Service (NHS).

IMPaCT therapy was refined following a staged model of behavioural therapies development [22–24]. The focus and key principles were guided by current literature regarding effective physical health interventions in mental health populations, which proposed integration of physical and mental health treatment, included Motivational Interviewing and Cognitive Behavioural Therapy approaches, and addressed a broader range of physical health and substance use targets [25–27], delivered over 9 months [28, 29].

IMPaCT therapy went through 3 stages of design and analysis: (1) development of therapy and training, and manual writing, incorporating consultation between experts in mental and physical health, substance use and diabetes; (2) piloting, evaluation and refining of the training package with clinicians and (3) Delphi process to reach consensus on the therapy model and manual. The Delphi process comprised an initial consultation followed by 2 rounds of follow-up questionnaire feedback from (i) expert therapist researchers (ii) clinician providers within Community Mental Health Teams(CMHTs) and (iii) psychosis service users.

Specifically, IMPACT Therapy [18] used motivational interviewing (MI) techniques to address lifestyle choices, with modules targeting the key areas of exercise, diet, tobacco smoking, alcohol use, cannabis use, other illegal substances and diabetes (where applicable), plus integration of cognitive behaviour therapy (CBT) skills to support behaviour change and mental health. To support the intervention, we published in book form a manual and a reference guide for clinicians and a handbook for service users [18]. We developed a four-day training programme for practitioners, encompassing skills and knowledge on physical health, substance use, health promotion, running groups, cognitive behavioural therapy and motivational interviewing. This was well attended and well received with an increase in mean self-rated knowledge scores from pre to post training on all core areas of training (physical health, substance use, running groups and using motivational interviewing).

### Study design and setting

We used a pragmatic multicentre, two arm, parallel cluster RCT design, integrated within community mental health teams (CMHTs) across five mental health NHS trusts in South London, Kent, Sussex, Somerset and Staffordshire, representing an urban to rural population. The study was planned and implemented in concordance with the Consolidated Standards of Reporting Trials (CONSORT) cluster trial extension standards [30] and details of method, measures, procedure, sample size calculation are described in the published protocol [31]. Ethical approval was obtained from the joint South London and Maudsley and the Institute of Psychiatry NHS Ethics Committee (REC Ref no 09/HO80/41). Colleagues with lived experience, both service users and carers, were involved throughout the research, from applying to funding to managing the steering group, to co-authoring this paper.

### Participants

Care co-ordinators in participating CMHTs who were permanently employed and had a minimum of four psychosis patients on their on their caseload who were eligible to participate in the study. The participants eligible for inclusion were aged between 18 and 65 years with a diagnosis of a psychotic disorder (ICD 10 diagnosis F20–29, F31.2, F31.5). Patients were excluded if they a) had a primary diagnosis of learning disability, b) had a pre-existing physical health problem that would independently impact on metabolic measures (as judged by medical investigators), c) were currently pregnant or less than 6 months post-partum or d) had a life threatening or terminal medical condition. We did not recruit from first episode psychosis services.

### Study procedure

The study procedure is published and available with open access [31], but will briefly be described. Recruitment of participants occurred in two waves; first, eligible community care coordinators were approached in a random sequence and invited to participate. Once a care co-ordinator gave informed consent, the patients from their caseload who met the inclusion criteria were likewise approached in a random order and sequentially invited to participate, until either 4 participants consented, or all eligible patients had been approached. Once baseline assessments were completed on all consenting patients in a care co-ordinator’s caseload, that care coordinator was randomised, stratified by borough (to allow for socio-economic differences between boroughs) using randomisation blocks of random sizes, to delivering IMPaCT Therapy or TAU alone to their own current patients (cluster). Researchers and the statistician remained blind to treatment allocation. Recruitment started on 01/03/2010 and the first date of randomization was 09.08.2010.

In the treatment arm, the intervention (IMPaCT Therapy) was provided by each patient’s usual community care coordinator who, within 3 months of randomisation, received the four-day IMPACT training course. Participating care coordinators were offered fortnightly supervision in IMPaCT Therapy throughout the subsequent 9-month intervention. Additionally, all care coordinators were offered a one-hour training session in best practice for physical health awareness to ensure more standardised treatment as usual (TAU).

### Outcome measures

Change in outcome was defined as difference from pre-randomisation (baseline) and i) at completion of the supervised intervention (12 months) and ii) 3 months after the end of treatment (15 months). Time windows to collect this data around the defined time points were established (minus 6 weeks/plus 4 weeks at 12 months and plus/minus 4 weeks for 15 month follow up). Data collected outside these times were recorded but only used for sensitivity analyses and not the main analysis.

#### Primary outcome

Primary outcomes were the physical and mental health component scores of the SF-36 [19] at 12 and 15 months.

A range of socio-demographic data was collected including age, sex, self-report ethnicity, marital status and current medications. Full details of measures are included in the protocol [31] In brief, secondary outcome measures included:

*Physical health measures:* Fasting blood samples including total, high (HDL) and low density lipoprotein (LDL) cholesterol, triglycerides, glycated haemoglobin (HBA1c) and C-reactive protein (CRP). Anthropometric measurements (waist circumference, body mass index (BMI), blood pressure) were also taken [31]. The International Diabetes Federation (IDF) criteria for Metabolic syndrome (MetS) were used to define abnormalities [32].

*Substance use measures*: Alcohol use was recorded using the Alcohol Use Disorders Identification Test (AUDIT) [33], tobacco use with the Nicotine Dependence Questionnaire [34], while use of cannabis and other illegal substances (opiates, methamphetamine, cocaine) was recorded using the Time Line Follow Back [35].

*Lifestyle measures*: Dietary pattern and physical activity were quantified according to the Dietary Instrument for Nutrition Education (DINE, [11]) and the short form International Physical Activity Questionnaire (IPAQ-SF, [36]) respectively.

*Mental Health status*: Participants completed the Positive and Negative Syndrome Scale (PANSS [37]), Global Assessment of Functioning (GAF) [38], SF-36 and Montgomery Asberg Depression Rating Scales (MADRS, [39]).

### Sample size

The power analyses were performed for the two subscales measures, physical and mental health components of SF-36 Quality of Life scale [19]. A sample size of 70 care coordinators each with an average of 4 patients (inflation factor 1.15 based on an assumed intraclass correlation of 0.05) and thus 280 patients, after allowing for 20% loss of care coordinator and an additional 30% loss of patients to follow up, was needed to detect a reduction of 5 points on both physical (d = 0.5) and mental health scale (d = 0.42) with at least 80% power using an alpha level of 0.05 and two-tailed assumptions.

### Statistical analysis

The primary statistical analyses were based on the intention to treatment principle and targeted at estimating the difference in the mean outcomes between participants randomised to HPI and TAU at the two post-treatment observation time points (12 and 15 months) using mixed effects models. Bias due to missing follow-up data was assessed by comparing baseline characteristics of those with and without complete data. In the two linear mixed effects models, physical and mental health component scores, respectively, at 12 months and 15 months constitute the dependent variable. “Treatment randomisation group”, “time (with two levels 12- and 15- months post-randomization)”, the interaction between “treatment group and time”, “centre”, and the “baseline values of physical and mental health component scores, respectively, are the fixed part of the model”. An unstructured covariance pattern model was used to model the dependency of the repeated observations of the same subject while care coordinator was included as a random factor to account for the dependency of the subjects within a cluster. Model assumptions were assessed by visual inspection of the residuals. Standardised effect sizes (using pre-randomisation variability for standardisation) are also reported.

#### Sensitivity analyses

Because about 15% of the observations were collected outside the time window we repeated the analyses using all available data as a sensitivity analyses. Demographic and clinical baseline characteristics were similar for both arms and we therefore did not perform any sensitivity analyses with baseline covariates [40]. Nor did we pre-specify any subgroup analyses to assess treatment effects to avoid increasing false positive and false negative findings due to inadequate power [41].

#### Handling of missing data

Models were rerun with predictors related to outcome missingness included as further covariates in the model. For the main outcomes, a second sensitivity analysis of missing outcome data, using multiple imputations by chained regression equations, was performed separately for each treatment group, using all available clinical and demographic scores.

#### Secondary outcomes

Secondary outcomes were analysed using the same methods as for the primary outcome. However, for all models the interaction between treatment group and time was not significant and was removed from the final analyses. Treatment effects are therefore estimates for both time points. Logistic mixed models were used for binary outcomes (such as smoking) and Poisson mixed models for count data (such as number of cigarettes per day).

### Role of the funding source

The funding body were not involved in the design, running or reporting of the trial.

## Results

**Fig. 1:**
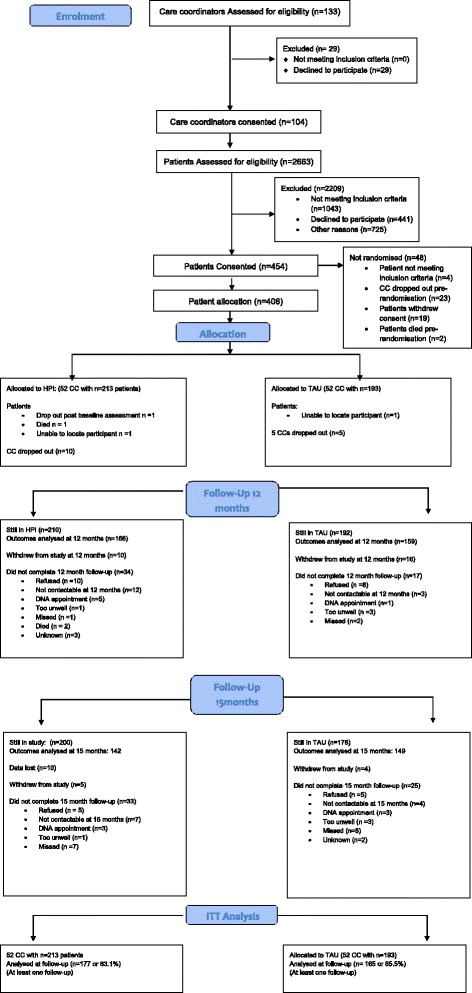
Consort diagram

In total, 104 care coordinators were randomised into the IMPACT health promotion intervention (HPI) and control group (TAU). Overall, 406 patients from randomised care coordinators were eligible and consented for the trial. Fifty-two care coordinators (with 213 patients, mean patients per care coordinator: 4.1, SD = 1.6, range 1–10) were randomized into HPI and 52 care coordinators (with 193 patients, mean patients per care coordinator 3.7, SD = 1.4, range 1–6) were randomised into TAU (Fig. 1 Consort diagram). Three hundred eighteen of the 406 patients (78.3%) attended the 12 months–follow-up and 301 (74.1%) attended 15 months follow-up. However, some of the patients who attended follow-up assessments were not seen within the required timeframe (10.5–13 months for 12 months and 14–16 for 15 months), so that the total sample size was reduced to 263 (64.8%) for 12 months (TAU: 132, HPI: 131) and 238 (58.6%) for 15 months (TAU: 114, HPI: 124). Three hundred and fourteen patients (77.3%, HPI: 75.6%, TAU: 79.3%) were seen at least at one time point within the time window and follow-up rates did not differ significantly between arms (12 months: chi2(1) = 1.81, *p* = 0.18, 15 months: chi2(1) = 1.80, p = 0.18). The required sample size based on the power analyses was achieved at both time points.

Pairwise comparison of baseline characteristics revealed that subjects who did not attend at 12 months follow-up were significantly younger (41.5 years (SD = 10.6)) than patients who did attend (44.9 years (SD = 9.88), (t(404) = 1.77, *p* = 0.005) and tended to have smaller baseline waist circumferences (103.6 cm (SD = 19.56) versus 107.6 cm (SD = 17.43)). Non-attendance at 12 months follow-up differed between centres (*p* = 0.01). No other demographic and clinical baseline characteristics differed significantly between attenders and non-attenders.

There were no significant differences in baseline characteristics between patients who attended within and outside the time windows.

Demographic and clinical characteristics at baseline are presented in Tables 1 and 3. Patients at baseline were on average 45 years old (range 22 to 66), predominantly male (55%) white (55%) and single (65%). Black patients were the main ethnic minority group (34%). The mean SF-36 Mental health score was 43.3 (range 9.6 to 67.7) and the Physical health score was 48.0 (18.8 to 68.3). Demographic and clinical baseline characteristics were similar for both arms except for some small difference in the relative number of patients per centre which was associated with small differences in the number of care coordinator per arm per centre (note; one centre had only one participating care co-ordinator).

**Table 1:** Demographic characteristics of participants at baseline for each trial arm and all patients combined

		TAU (*N* = 193, 117 male)		HPI (*N* = 213, 117 male)		Total (*N* = 406, 234 male)	
		Mean (SD) or No.	Range or %	Mean (SD) or No.	Range or %	Mean (SD) or No.	Range or %
Age at baseline	Years	44.65 (10.17),	22.95–65.96	43.76 (10.09)	21.89–65.94	44.18 (10.12)	21.89–65.96
Education	None	47	24.6	61	28.6	108	26.7
	GSCE/O-Level/Level 1 or 2 NVQ	68	35.6	91	42.7	159	39.4
	A-Level/Level 3/NVQ	33	17.3	33	15.5	66	16.3
	Bachelor Degree/Graduate Certificate/Diploma or post-graduate qualification	43	22.5	28	13.1	71	17.6
	Total	191	100	213	100	404	100
Ethnicity	White	100	52.1	122	57.8	222	55.1
(4 groups)	Black	68	35.4	69	32.7	137	34.0
	Asian	8	4.2	7	3.3	15	3.7
	Mixed and other	16	8.3	13	6.2	29	7.2
	Total	192	100	211	100	403	100
Relationship status	Single	118	61.1	139	66.2	257	63.8
	Married/co-habiting	29	15	27	12.9	56	13.9
	Steady relationship	20	10.4	15	7.1	35	8.7
	Divorced/Separated/Widowed	26	13.4	29	13.9	55	13.7
	Total	193	100	210	100	403	100
Borough	Croydon	33	17.1	25	11.7	58	14.3
	Lambeth	23	11.9	21	9.9	44	10.8
	Lewisham	34	17.6	50	23.5	84	20.7
	Southwark	42	21.8	41	19.2	83	20.4
	Greenwich	20	10.4	25	11.7	45	11.1
	Bromley	9	4.7	10	4.7	19	4.7
	Bexley	12	6.2	11	5.2	23	5.7
	East Sussex	12	6.2	11	5.2	23	5.7
	Somerset	8	4.1	9	4.2	17	4.2
	South Staffordshire	0	0	10	4.7	10	2.5
	Total	193	100	213	100	406	100

*TAU* treatment as usual, *HPI* IMPACT therapy (Health Promotion Intervention)

### Primary outcome analyses

**Fig. 2:**
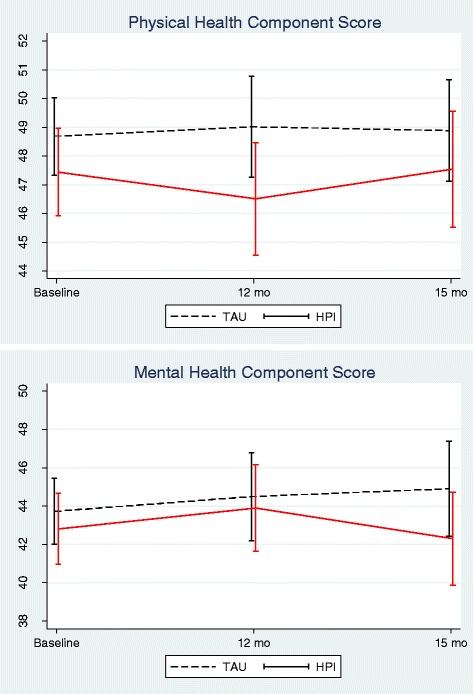
Mean Physical health and mental health component score (95% Confidence interval) at baseline, 12 and 15 months follow-up for Treatment as usual (TAU) and Health intervention programme group (HPI)

The mixed effect model analyses revealed no significant treatment effect for change in either Physical or Mental health scores between TAU and IMPaCT at either 12 or 15 months (Fig. 2, Table 2). Estimated treatment effect sizes were small (Physical health score, d = −0.17 at 12 months and −0.09 at 15 months, Mental health score, d=: 0.03 at 12 months and −0.05 at 15 months). There were no significant differences in effect between centres. After removing the non-significant interactions between treatment arm and time, patients in the treatment group showed a non-significant decrease of 1.4 (95% CI: -3.2 to 0.41, *p* = 0.13, d = −0.12) in physical health scores and 0.13 (95% C.I -2.49 to 2.24), *p* = 0.92, d = −0.01) in mental health scores compared to the patients in the control group after adjusting for baseline differences.

**Table 2:** Results of the mixed effect linear model for main outcomes (physical and mental health score) with treatment arm at 12 months or 15 months

	SF-36 Physical Health Score				SF-36 Mental Health Score			
Variable	Coefficient	(95% CI)	z	P	Coefficient	(95% C.I.)	z	p
Arm (HPI = 1)	−1.77	(−3.88 to 0.34)	−1.65	0.10	0.37	(−2.41 to 3.15)	0.26	0.79
Time (15 mo = 1)	−0.15	(−1.73 to 1.44)	−0.18	0.86	0.22	(−1.75 to 2.18)	0.22	0.83
Arm x Time	0.77	(−1.49 to 3.03)	0.67	0.51	−1.05	(−4.15 to 2.04)	−0.67	0.51
Borough	Chi2(9) = 8.37, *p* = 0.50				chi2(9) = 8.82, *p* = 0.45			
Pairwise comparison	Coefficient	[95% CI]	z	P			z	P
12 mo: HPI vs TAU	−1.77	(−3.88 to 0.34)	−1.65	0.10	0.37	(−2.41 to 3.15)	0.26	0.79
15 mo: HPI vs TAU	−1.00	(−3.15 to 1.14)	−0.92	0.36	−0.68	(−3.56 to 2.19)	−0.47	0.64
Group HPI: 15 mo vs 12 mo	−0.15	(−1.73 to 1.44)	−0.18	0.86	0.22	(−1.75 to 2.18)	0.22	0.83
Group TAU: 15 mo vs 12 mo	0.62	(−1 to 2.25)	0.75	0.45	−0.84	(−3.24 to 1.56)	−0.68	0.49
Random effect care coordinator	1.06	(0.07 to 15.29)			2.17	(0.72 to 6.56)		

Results of the mixed effect linear model for main outcomes (physical and mental health score) with treatment arm, time (12 months or 15 months), the interaction between treatment arm and time, borough and baseline values of outcome. Pairwise comparisons show treatment effect estimates at 12 and 15 months and changes from 12 to 15 months within each treatment arm. Care coordinator was included as a random factor to account for the dependency of observations with care coordinator. The dependency of repeated observations within individuals was modelled by estimating the variance–covariance structure of the residuals

*TAU* treatment as usual, *HPI* IMPACT therapy (Health Promotion Intervention)

#### Sensitivity analyses

Including data obtained outside the observation period resulted only in minor changes and did not alter the conclusion. Similarly, including age as a predictor of missingness and a multiple imputation analyses for missing data resulted only in minor changes and did not alter the conclusion that there was no significant treatment effect at any time point (Models with age (raw figures) included: physical health score: d = −1.52 (95% CI -3.3 to 0.26, *p* = 0.094, Mental health score d = −0.09 (95% C.I. -2.45 to 2.27), *p* = 0.94; Multiple imputation: Treatment effect Physical health score, d = −0.78 (95% C.I.: -3.76 to 2.20), *p* = 0.61, mental health score, d = −0.27 (95% C.I.: -3.82 to 0.27), *p* = 0.90; all analyses without treatment x time interaction).

### Secondary outcomes

Table 3 shows estimated treatment effects of the secondary clinical outcomes at 12 and 15 months follow-up. The only significant difference in any of the secondary outcomes at either 12 or 15 months was in HDL Cholesterol, which improved more with IMPACT therapy than in the TAU group (Treatment effect (95% CI); 0.085 (0.007 to 0.16); *p* = 0.034, Cohen’s d = 0.2). The other effect sizes (Cohen’s d) for continuous outcomes ranged from −0.15 to 0.19. Twenty-eight patients (13.15%) in the HPI group showed a serious adverse event (SAE) while 21 (10.88%) of the patients in TAU showed an SAE. This difference is not significant (chi^2^(1) = 0.41, *p* = 0.52).

**Table 3:** Clinical characteristics of participants at baseline for all participants and changes over time for each arm separately at baseline, 12 months and 15 months follow-up and estimated pooled treatment effects (95% confidence intervals), *p* value of test and effect size Cohen’s d for continuous outcomes

Variable	Combined		Treatment as Usual (TAU)						IMPACT Therapy (Health Promotion Intervention (HPI))						Mixed model results		
	Baseline		Baseline		12 months		15 months		Baseline			12 months		15 months			
	N	Mean(SD)/N(%)	N	Mean(SD)/N(%)	N	Mean(SD)/N(%)	N	Mean(SD)/N(%)	N	Mean(SD)/N(%)	N	Mean(SD)/N(%)	N	Mean(SD)/N(%)	Treatment effect (95% C.I.)	*p* value	Cohen’s d
SF-36 PCS	406	48.03 (10.54)	193	48.68 (9.58)	132	49.01 (10.33)	114	48.88 (9.62)	213	47.44 (11.32)	127	46.51 (11.24)	117	47.54 (11.14)	−1.40 (−3.20 to 0.41)	0.13	−0.13
SF-36 MCS	406	43.25 (13.02)	193	43.72 (12.22)	132	44.5 (13.47)	114	44.91 (13.42)	213	42.81 (13.73)	127	43.9 (12.97)	117	42.3 (13.42)	−0.13 (−2.49 to 2.24)	0.92	0.01
BMI <100 only	380	31.17 (7.52)	177	31.79 (7.49)	124	32.29 (8.01)	102	33.6(9.61)	203	30.63 (7.52)	120	30.51 (7.38)	103	30.04 (7.67)	−0.45 (−1.32 to 0.42)	0.31	−0.06
Waist (cm)	378	106.81 (17.94)	178	107.39 (16.38)	124	109.25 (16.52)	108	110.96 (16.62)	200	106.29 (19.24)	121	106.05 (16.44)	111	104.22 (16.21)	−1.83 (−3.82 to 0.17)	0.07	−0.1
Smoker	405	253 (62.5%)	193	112 (58)	132	80 (62.1)	113	64 (56.6)	212	141 (66.5)	127	81 (63.8)	116	74 (63.8)	1.05 (0.37 to 3.03)	0.93	N/A
Cigarettes/day (if smoker)	244	18.30 (11.54)	108	18.27 (9,75)	80	18.06 (11.04)	61	17.31 (10.67)	136	18.32 (12.83)	81	19.79 (15.49)	71	18.97 (15.59)	0.1 (−0.33 to 0.53)	0.65	N/A
Cannabis user	405	50 (12.3)	193	26 (13.5)	158	21 (13.3)	148	12 (8.1)	212	24 (11.3)	160	21 (13.1)	153	21 (13.7)	1.40 (0.51 to 3.93)	0.50	N/A
Cocaine use	405	7 (1.7)	193	3 (1.5)	158	2 (1.3)	148	1 (0.7)	212	4 (1.9)	160	0 (0)	153	1 (0.9)	–	–	–
HBA1c (%)	303	40.49 (8.17)	148	40.93 (8.34)	91	41.77 (9.16)	85	40.79 (10.69)	155	40.06 (8)	80	38.6 (9.2)	86	39.28 (10.48)	−0.32 (−1.49 to 0.86)	0.59	−0.04
Cholesterol	289	5.16 (1.46)	136	5.07 (1.11)	86	4.86 (1.12)	80	4.70 (1.32)	153	5.23 (1.72)	80	4.96 (1.02)	85	4.81 (1.00)	0.01 (−0.28 to 0.30)	0.93	0.01
HDL cholesterol	306	1.25 (0.43)	137	1.28 (0.35)	86	1.25 (0.34)	80	1.19 (0.34)	153	1.23 (0.5)	80	1.30 (0.30)	85	1.30 (0.35)	0.085 (0.007 to 0.16)	0.034	0.20
LDL cholesterol	290	3.06 (1.01)	137	3.03 (1)	86	2.77 (1.07)	80	2.73 (1.11)	153	3.08 (1.01)	80	2.9 (0.96)	85	2.75 (0.95)	0.06 (−0.11 to 0.24)	0.49	0.06
Triglycerides	307	2.08 (1.7)	150	2.12 (1.67)	91	2.23 (1.97)	86	2.03 (1.38)	157	2.04 (1.74)	82	1.82 (1.4)	90	2.09 (2.47)	0.01 (−0.37 to 0.4)	0.95	0.01
C-Reactive Protein (CRP)	238	5.65 (7.91)	124	5.46 (7.02)	24	6.59 (6.35)	17	4.98 (5.49)	114	5.85 (8.81)	20	4.55 (3.79)	11	4.2 (4.78)	0.76 (−1.25 to 2.77)	0.46	0.1
Hypertension	380	246 (64.7%)	178	110 (61.8%)	125	82 (65.6%)	106	67 (63.2%)	202	136 (67.3%)	120	76 (63.3%)	112	68 (60.7%)	0.71 (0.38 to 1.36)	0.31	N/A
DINE saturated fat score	393	31.69 (12.74)	186	31.88 (12.54)	125	32.47 (14.33)	110	31.76 (14.13)	207	31.52 (12.95)	123	31.83 (13.23)	110	31.19 (12.61)	−0.19 (−2.82 to 2.44)	0.89	−0.01
Audit	270	5.8 (6.09)	127	5.4 (5.77)	79	5.05 (5.54)	68	4.76 (4.83)	143	6.15 (6.35)	84	6.98 (6.46)	77	7.43 (6.69)	0.19 (−0.02 to 0.39)	0.07	0.19
IPAQ	99	3197.74 (3701.23)	47	3472.78 (3841.4)	36	2543.28 (3554.38)	28	2011.84 (2738.3)	52	2949.14 (3589.07)	32	1540.72 (1563.33)	23	2695.74 (2764.05)	−0.08 (−0.4 to 0.25)	0.65	−0.02
MADRS	403	10.99 (9.46)	193	11.05 (9.33)	132	10.23 (8.97)	114	9.92 (8.8)	210	10.93 (9.59)	127	10.87 (9.56)	115	12.03 (9.99)	0.49 (−1.23 to 2.22)	0.58	0.05
GAF	402	59.33 (13.19)	193	60.88 (13.38)	130	55.56 (12.64)	114	53.59 (11.01)	209	57.9 (12.87)	126	53.01 (11.03)	117	52.63 (11.42)	−0.7 (−3.44 to 2.03)	0.62	−0.05
PANSS (total)	399	51.38 (14.14)	191	51.63 (14.46)	131	50.08 (12.74)	114	49.4 (14.8)	208	51.14 (13.86)	127	51.24 (13.44)	115	50.8 (13.77)	1.37 (−1.23 to 3.96)	0.3	0.1

Presented descriptive statistics are mean (SD) for continuous variables and N (%) for categorical data. Statistics for changes over time within treatment arm and treatment effects are based on patients with follow-up data collected within defined time window

Hypertension, smoking and cannabis use were binary variables (yes or no) and a logistic model was used. Treatment effects are odds ratios. Number of cocaine users was too small to fit a logistic model

Number of cigarettes was modelled using a Poisson distribution. Treatment effects are incidence rate ratios. Only models with random intercept for care coordinators and id could be fitted for logistic and Poisson models. A model for saturated fat categories could not be estimated

Care coordinators (CC) allocated to the HPI arm of the trial saw their patients for an average of 8.44 (SD 5.3; range 0–25) sessions over the course of the trial. These sessions lasted on average (50.7 min (SD 27.1). Of this, a median of 20 min was dedicated to the HPI rather than usual care. Minimum adherence to the intervention protocol was defined as at least 6 sessions. In interpreting the data, we later defined the minimum session duration as 30 min in addition to routine care; this was delivered by 9 (17.3%) out of the 52 HPI care coordinators to 19 of their patients. Forty-seven patients (22.0%), under the care of 19 care coordinators (36.5%) in the treatment group, either attended at least six 30-min sessions of IMPaCT Therapy in addition to their usual contact time or had the same therapy duration (180 min) spread over more than 6 sessions. These patients had a non-significant decrease of 2.45 (95% CI: -5.51 to 0.62, d = −0.23, *p* = 0.12) in physical health scores and 0.84 (95% C.I -4.44 to 2.76), d = −0.02, *p* = 0.65) in mental health scores compared to the patients in the control group over the 12 months, after adjusting for baseline differences. They had a significantly greater reduction in waist circumference over 12 months than did controls (−4.20 cm (95% C.I. -7.18 to – 1.23), *p* = 0.006). All other assessed variables remained non-significant, but only continuous outcomes could be assessed due to the smaller sample size.

## Discussion

### Statement of principal findings

The current study, the largest pragmatic trial of a MI/CBT HPI intervention in the UK, delivered by care coordinators to their own patients with psychosis and integrated into secondary care practice, found the addition of IMPaCT therapy had no significant effect on our primary outcomes of PCS and MCS quality of life scores. In addition, there was little evidence that IMPaCT therapy delivered by CCs improved cardiovascular risk indicators, substance use or mental health measures compared to TAU alone. The only advantage observed with IMPaCT therapy over TAU at the intention to treat level was in the relatively small effect on HDL cholesterol, which must be interpreted with caution, in the context of multiple testing (see Table 3). Higher HDL levels are linked to cardiovascular health and respond to dietary change and exercise.

### Strengths of the study

This is the first RCT investigating an integrated health promotion intervention designed to be used in routine clinical care and implemented by the patient’s usual care coordinator. The main strength of this trial is that it was a pragmatic study set in the NHS, designed to be as representative as possible. The NHS delivers the bulk of healthcare to people with psychosis, largely in secondary care, although if the course of illness is stable, people are discharged to primary care. Community secondary care treatment is led by care co-ordinators based in Community Mental Health Teams (CMHTs) who see their patients in various settings; it was they who were invited to take part in the trial and, if randomised to the treatment arm, deliver the intervention. The HPI was thus accessible to the greatest possible proportion of people with psychosis receiving secondary care, many of whom may not wish, or may be too unwell to attend add-on or group interventions. Thus it attempted to meet the needs of this very hard-to-reach group with high rates of cardiometabolic disease [7] by enhancing routine care. The study recruited to target from a diverse multi-ethnic sample of people with psychosis and had good follow-up rates. There was no difference in dropout between the active and control arms of the study, although those who did not attend at 12 months follow-up were younger. To limit multiple testing and due to the small numbers in some sub-categories we did not do sub-analyses of outcomes by ethnicity, age groups or sex.

The relationship between borough and non-attendance at 12 months follow-up is likely to be multifactorial.

This study attempted to influence lifestyle choices and substance use, which affect both physical and mental health. To date, few large-scale long-term RCTs have attempted to improve health in its widest sense in people with psychosis, with most concentrating on a single target, usually weight, and interventions largely run by therapists outside of the usual clinical team. Even where the work has been co-located, they have been separate to usual care [15, 17]. Therefore, our integrated personalised approach, conducted by the core mental health clinician adapting to the patient’s individual needs, attempted to address some of these concerns by maximising representativeness of the population studied and avoiding a piecemeal approach to behavioural change. Further, our choice of the SF-36 PCS and MCS scores as primary outcome measure reflected our hypothesis that the lifestyle interventions would lead to a general and perceptible improvement in both mental and physical health. Our study, like the recent Change RCT in Denmark [17], demonstrates the challenges of reducing cardiovascular risk among those with established psychosis.

### Limitations of the study

There are a number of contextual factors that may have influenced the outcome of the trial. First, this study was funded before the promotion of improved physical health and lifestyle choices among people with psychosis was rising on the national and local NHS agenda. Since that time in the UK alone, the ‘parity of esteem’ movement to improve physical healthcare for people with psychosis was successfully launched by the Royal College of Psychiatrists, NICE published guidance on physical health and the Quality and Outcomes Framework (QOF) offered payment to GPs to monitor and advise on physical health outcomes and lifestyle choices (e.g. encouraging patients to stop smoking). It is likely that participants in both treatment arms benefited from the concerted efforts to improve the very outcomes we were targeting within the study, although we did not demonstrate a significant improvement in either arm over the time frame of the study. Secondly, multiple re-organisations among the participating hospital trusts affected continuity of care. Staff turnover was slightly greater in the treatment than control arms, although this was not statistically different. In this context however, it is interesting to note that where patients remained with the same trained care coordinator throughout the trial, there were indications that the intervention may result in significantly lower cholesterol levels and more exercise in the treatment arm than in controls. Thirdly, given the integration with routine care, it was not possible to control for conditions such as minutes per session. Despite pilot development work and regular supervision being offered by research therapists, care co-ordinators struggled to deliver 6 or more sessions of 30 min each of the HPI in addition to routine care, with HPI sessions lasting a median of 20 min. This highlights the recognised challenges for care co-ordinators delivering targeted psychosocial interventions to people with psychosis in a busy secondary care environment. The proportion of care co-ordinators who delivered the minimum dose (17–36% dependent on the measure taken), was in keeping with Harding et al. (under review [42]) who found that only 25% of care co-ordinators delivered a brief CBT-based therapy despite adequate training and supervision, compared to 62% of assistants without case management responsibilities. This is also consistent with other studies that have found that care co-ordinators struggle to implement therapy alongside case management [43, 44]. Where participants received the intervention of at least 180 min in addition to routine care, the significantly greater reduction in waist circumference than in controls was of a clinically significant degree, suggesting a dose effect once a minimum therapy time was delivered.

A further consideration is that our modular interventions were broad and participants self-selected the target behaviours to be focused on. It is possible that more structured interventions targeted at specific health behaviours, such as those described elsewhere [15, 16, 45] may be better placed to demonstrate statistically significant improvement in defined aspects of the physical health of this population. However, for the purposes of routine care, a more long term, sustainable and integrated approach to global health behaviours is urgently required.

### Clinical and service implications

This is the first trial to address the question of whether a health promotion intervention delivered by the core mental health clinician as part of enhanced routine care can achieve greater clinically significant health gains in comparison to treatment as usual. The answer is that this integrated approach did not appear to match the modest health gains (usually measured by weight loss) seen by adding group interventions to usual care – although it would be of interest to compare representativeness of samples between this integrated (and thus very accessible) approach and add-on interventions. Nor was additional time for the intervention regularly delivered when rolled out across community mental health teams. Thus the message to service planners is clear and important – by itself focussed training and supervision provision for front-line mental health workers does not appear to be enough to change therapeutic practice sufficiently to reverse cardiometabolic risk indicators in their patients with established psychosis. However our data suggests that continuity of care and protected time to deliver HPI work may well enhance outcomes, an important practical point in today’s rapidly evolving health services.

A recent qualitative review of reviews [46] considering non pharmacological interventions seeking to reduce cardiometabolic risk concluded that interventions with multiple components, personalized, with more frequent face-to-face contact, and professionally trained treatment providers are associated with better outcomes. Given that our trial showed little effect at the intention to treat level of training and offering supervision to CMHT staff in delivering a HPI, it may be that widening mental health teams to include specialists in exercise and nutrition is necessary, as in the Keeping the Body in Mind preventative programme in Sydney [47]. This will have cost implications for service providers and thus may introduce further barriers to accessing care, pending evaluation of the cost-effectiveness of such models. Our results strongly support the notion that prevention of emergence of cardiometabolic risk factors in early psychosis is key, as reversing these in established illness is extremely difficult [17]. Of note, only people with established illness were included in our study.

Focussing on reducing BMI and waist circumference is notoriously challenging [46]. Future multicomponent interventions might wish to consider cardiorespiratory fitness (the ability of the circulatory and respiratory systems to supply oxygen to working muscles during sustained physical activity) as the primary outcome, since there is mounting evidence that ‘fitness’ is also an important predictor of mortality [48].

## Conclusions

The current paper is the first randomised control trial investigating a health promotion intervention designed to be used in routine clinical care and implemented by the patient’s usual care coordinator. Our study found that training and supervising care co-ordinators to work with their own patients with psychosis to change lifestyle choices is not in itself sufficient to effect clinically or statistically significant improvement in quality of life or overall cardiovascular risk in people with established psychosis, although there was an effect on waist circumference when the minimum dosage was delivered. Given the need to reduce the life expectancy gap between people with psychosis and the general population, the search across health systems for more effective, deliverable, affordable and sustainable interventions remains a priority.

## Abbreviations

AUDIT: Alcohol Use Disorders Identification Test
BMI: Body mass index
CBT: Cognitive behaviour therapy
CC: Care Coordinator
CMHTs: Community mental health teams
CONSORT: Consolidated Standards of Reporting Trials cluster trial extension standards
CRP: C-reactive protein
CVD: Cardiovascular disease
DINE: Dietary Instrument for Nutrition Education
GAF: Global Assessment of Functioning
HBA1c: Glycated haemoglobin
HDL: High density lipoprotein
HPI: Health Promotion Intervention
IDF: International Diabetes Federation
IMPACT: Improving health and reducing substance use in established psychosis
IPAQ: International Physical Activity Questionnaire
LDL: Low density lipoprotein
MADRS: Montgomery Asberg Depression Rating Scales
MCS: Mental component of the Short form (SF)-36
MetS: Metabolic syndrome
MI: Motivational Interviewing
PANSS: Positive and Negative Syndrome Scale,
PCS: Physical component of the Short form (SF)-36
RCT: Randomized controlled trial
SF: Short form −36
TAU: Treatment as usual
TAU: Treatment as usual
